# Historical Trends and Epidemiological Indicators of Congenital Syphilis in São Paulo, Brazil: 1986-2023

**DOI:** 10.1590/0037-8682-0113-2025

**Published:** 2025-12-19

**Authors:** Mariana Rebelo Matos, Gustavo Yano Callado, Edward Araujo, Karina Felippe Monezi Pontes

**Affiliations:** 1 Hospital Ipiranga, Serviço de Ginecologia e Obstetrícia, São Paulo, SP, Brasil.; 2 Hospital Israelita Albert Einstein, Faculdade Israelita de Ciências da Saúde Albert Einstein, São Paulo, SP, Brasil.; 3 Universidade Federal de São Paulo, Escola Paulista de Medicina, , Departamento de Obstetrícia, São Paulo, SP, Brasil.; 4 Universidade Municipal de São Caetano do Sul, Disciplina de Saúde da Mulher, São Caetano do Sul, SP, Brasil.

**Keywords:** Pregnancy complications, Infectious, Congenital syphilis, Perinatal care

## Abstract

**Background:**

This study aimed to present historical trends and key epidemiological indicators of congenital syphilis in São Paulo, Brazil, from 1986 to 2023.

**Methods:**

This retrospective cross-sectional study used secondary data.

**Results:**

A total of 56,547 cases and 690 deaths from congenital syphilis were reported. The incidence rate steadily increased over time. The number of stillbirths and miscarriages also rose. Most cases of maternal syphilis infection were identified during prenatal care, and the rate of inadequate maternal treatment remained high. Partner treatment led to modest improvements.

**Conclusion:**

Despite improvements in prenatal care coverage and earlier diagnosis, the persistent rise in congenital syphilis incidence and mortality highlights ongoing gaps in maternal and partner treatment.

Syphilis is a systemic infectious disease caused by the bacterium *Treponema pallidum*, which belongs to the spirochete family. This pathogen has a high capacity for immune evasion, and its clinical manifestations are wide-ranging, affecting various tissues. Transmission occurs primarily through sexual contact and vertical transmission, and less frequently through direct contact with syphilitic lesions during delivery or via blood transfusion^1^ ^,^ ^2^. 

Congenital syphilis results from the vertical transmission of *Treponema pallidum* through the transplacental route. This transmission can occur at any stage of maternal infection; however, the bacterial load is higher during the primary and secondary stages, making transmission more likely. In addition, the risk of fetal infection increases with advancing gestational age owing to increased placental permeability. Congenital syphilis can lead to physical, sensory, and developmental sequelae in affected newborns, as well as adverse pregnancy outcomes^3^ ^,^ ^4^. 

Between 2017 and 2022, 43 cases (21.2%) of congenital syphilis were confirmed at a tertiary hospital in Mexico. According to the clinical stage of maternal syphilis, 119 women (66.1%) were in the late latent phase, 49 (27.2%) in the early latent phase, seven (3.9%) in the secondary stage, and five (2.8%) in the primary stage^5^. In a recent epidemiological study in Brazil from 2012 to 2021, 192,055 cases of congenital syphilis were reported, with the southeastern region showing the highest incidence rate of 7.25 cases per 1,000 live births^2^. An increase in incidence was observed across all regions, and although national rates increased, no statistically significant difference was found between the mean rates of the two evaluated periods. Between 2013 and 2018, in the city of Campo Grande, the capital of Mato Grosso do Sul in the Midwest region, the cumulative detection rate of gestational syphilis was 174.3 cases per 1,000 live births, whereas the cumulative incidence of congenital syphilis was 47.7 cases per 1,000 live births^6^.

Globally, congenital syphilis remains a persistent public health challenge, with increasing incidence reported in various countries despite prenatal screening programs. In Brazil, the disease has shown a consistent upward trend, particularly in the southeastern region. However, few studies have examined long-term epidemiological changes over nearly four decades, limiting our understanding of progress or stagnation in disease control. This study seeks to fill this gap by analyzing data from São Paulo, the most populous Brazilian state.

This study aimed to present the historical epidemiological profile of congenital syphilis in São Paulo, Brazil, between 1986 and 2023. It addresses a knowledge gap regarding the long-term temporal trends of congenital syphilis by providing a comprehensive analysis that integrates incidence, mortality, and prenatal care indicators.

This longitudinal ecological study was based on aggregated secondary data from reported cases of congenital syphilis in São Paulo between 1986 and June 2023, representing the most recent data available. Data were extracted from the Information System for Notifiable Diseases (SINAN/DATASUS) and are publicly available from the Brazilian Ministry of Health. As the study used secondary public domain data without individual identification, it was exempt from ethical approval in accordance with Resolution CNS No. 510/2016 of the Brazilian National Health Council.

A reported case of congenital syphilis was defined according to the criteria established by the Brazilian Ministry of Health. The incidence rate was calculated as the number of reported cases per 1,000 live births per year, and the mortality rate as the number of deaths from congenital syphilis per 100,000 live births per year. Live birth data were obtained from the SINASC system.

The variables analyzed included the absolute number of congenital syphilis cases, incidence rate of congenital syphilis (IRCS), and mortality rate of congenital syphilis (MRCS). In addition, adverse pregnancy outcomes associated with congenital syphilis were assessed. The analysis also included prenatal care-related variables such as the timing of maternal diagnosis, treatment adequacy, and partner treatment. The timing of the maternal diagnosis was categorized as during prenatal care, at delivery or curettage, or after delivery.

The outcomes were categorized as live birth, miscarriage, stillbirth, death from congenital syphilis, death from other causes, or unknown. Maternal diagnosis timing was grouped as during prenatal care, at delivery/curettage, after delivery, not performed, or unknown.

Adequate maternal treatment was defined as the administration of penicillin appropriate for the clinical stage of syphilis, completed at least 30 days before delivery, with documented clinical and serological responses. Treatment was considered inadequate if these criteria were not met, the regimen was incomplete, treatment was administered <30 days before delivery, or non-penicillin antibiotics were used. Cases with no treatment were categorized as “not performed.”

Quantitative data were transcribed and organized in Excel 2019 (Microsoft Corp., Redmond, WA, USA), and analyses were conducted using R software version 4.5.1 (R Development Core Team, Vienna, Austria, 2025). Temporal trends in incidence and mortality were evaluated using joinpoint regression analysis, which identified statistically significant changes in trend slopes over time.

Between January 1986 and June 2023, 56,547 reported cases of congenital syphilis and 690 related deaths were recorded in São Paulo. From 1986 to 2022, there was a progressive and sustained increase in both the absolute number of cases and IRCS (Figure 1A). Between 1986 and 1992, the number of cases remained consistently low, not exceeding 100 cases per year, with an IRCS of 0.0. Beginning in 1993, there was a gradual increase, with cases exceeding 300 and IRCS values reaching 0.5 per 1,000 live births. This upward trend continued with some fluctuations but followed a general pattern of increase. A marked acceleration was observed in 2010, when both the number of cases and the IRCS rose more sharply, from 1,179 cases and an IRCS of 2.0 per 1,000 live births in 2010 to 2,976 cases and an IRCS of 4.8 per 1,000 live births in 2014. In the subsequent period (2015-2022), the upward trend persisted, culminating in 4,536 reported cases and an IRCS of 8.9 per 1,000 live births by 2022. 

**FIGURE 1:**
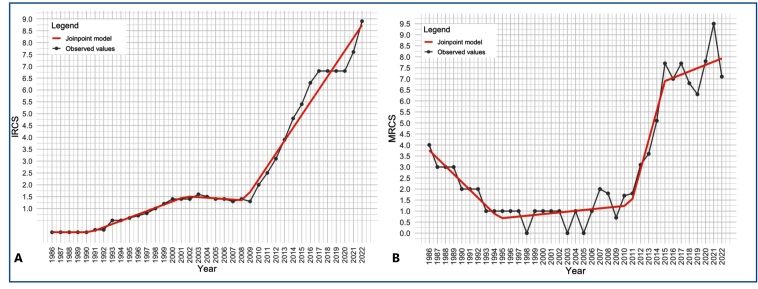
Annual reported incidence **(A)** and mortality **(B)** rates of congenital syphilis per 1,000 and 100,000 live births, respectively, with Joinpoint regression trend lines. São Paulo, Brazil, 1986-2022. **Source:** SINAN/DATASUS - Brazilian Ministry of Health.

The temporal distribution of deaths and MRCS between 1986 and 2022 (Figure 1B) showed two distinct periods. From 1986 to the early 2000s, the number of deaths steadily declined from 29 deaths and an MRCS of 4.0 in 1986 to <10 deaths per year, with MRCS values at or near 1.0 per 100,000 live births between 1993 and 2007. However, from 2012 onward, there was a marked increase in both absolute deaths and MRCS. Notably, the MRCS rose from 3.1 per 100,000 live births in 2012 to 7.7 per 100,000 in 2015, peaking at 9.5 per 100,000 in 2021, when 50 deaths were recorded. Although year-to-year variations were observed, mortality indicators demonstrated a consistent upward trend after 2012.

Among pregnancies affected by congenital syphilis, live births remained the most frequent outcome, ranging from 85.2% to 89.8% between 2011 and 2023 (Table 1). Deaths from congenital syphilis fluctuated between 0.7% and 1.4%, peaking in 2015 and again in 2020-2021. Miscarriages increased from 4.3% in 2011 to 7.7% in 2019-2021, before declining slightly to 6.7% by 2023. Stillbirths varied over time, reaching a maximum of 5.7% in 2014 and stabilizing between 3.6% and 4.2% in subsequent years. Deaths from other causes and outcomes classified as unknown remained below 2.2% throughout the study period.

**TABLE 1:** Timing of maternal syphilis diagnosis, prenatal care attendance, classification of maternal syphilis treatment, partner treatment status, and perinatal outcomes in the state of São Paulo, Brazil, 2011-2023

	2011 (n = 1,506)	2012 (n = 1,926)	2013 (n = 2,397)	2014 (n = 2,976)	2015 (n = 3,394)	2016 (n = 3,807)	2017 (n = 4,151)	2018 (n = 4,096)	2019 (n = 3,980)	2020 (n = 3,732)	2021 (n = 4,009)	2022 (n = 4,536)	2023 (Jan -Jun) (n = 1,917)	Total (n = 42,427)
Timing of maternal diagnosis														
During prenatal care	781 (51.9%)	956 (49.6%)	1,261 (52.6%)	1,574 (52.9%)	1,950 (57.5%)	2,390 (62.8%)	2,720 (65.5%)	2,319 (56.6%)	2,470 (62.1%)	2,318 (62.1%)	2,610 (65.1%)	3,023 (66.6%)	1,294 (67.5%)	27,489 (59.7%)
At delivery/curettage	591 (39.2%)	807 (41.9%)	955 (39.8%)	1,126 (37.8%)	1,158 (34.1%)	1,126 (29.6%)	1,185 (28.5%)	1,503 (36.7%)	1,281 (32.2%)	1,176 (31.5%)	1,153 (28.8%)	1,282 (28.3%)	530 (27.6%)	15,478 (33.6%)
After delivery	97 (6.4%)	128 (6.6%)	128 (5.3%)	188 (6.3%)	184 (5.4%)	176 (4.6%)	88 (2.1%)	111 (2.7%)	103 (2.6%)	99 (2.7%)	103 (2.6%)	95 (2.1%)	32 (1.7%)	1,583 (3.4%)
Not performed	11 (0.7%)	12 (0.6%)	12 (0.5%)	15 (0.5%)	10 (0.3%)	25 (0.7%)	24 (0.6%)	29 (0.7%)	26 (0.7%)	25 (0.7%)	25 (0.6%)	21 (0.5%)	20 (1.0%)	266 (0.6%)
Unknown	26 (1.7%)	23 (1.2%)	41 (1.7%)	73 (2.5%)	92 (2.7%)	90 (2.4%)	134 (3.2%)	134 (3.3%)	100 (2.5%)	114 (3.1%)	118 (2.9%)	115 (2.5%)	41 (2.1%)	1,203 (2.6%)
Prenatal care attendance														
Yes	1,115 (74%)	1,345 (69.8%)	1,712 (71.4%)	2,232 (75%)	2,634 (77.6%)	3,035 (79.7%)	3,429 (82.6%)	3,262 (79.6%)	3,241 (81.4%)	3,067 (82.2%)	3,372 (84.1%)	3,822 (84.3%)	1,603 (83.6%)	36,661 (79.7%)
No	346 (23%)	528 (27.4%)	584 (24.4%)	634 (21.3%)	619 (18.2%)	642 (16.9%)	585 (14.1%)	661 (16.1%)	592 (14.9%)	521 (14%)	500 (12.5%)	608 (13.4%)	250 (13%)	7,754 (16.8%)
Unknown	45 (3%)	53 (2.8%)	101 (4.2%)	110 (3.7%)	141 (4.2%)	130 (3.4%)	137 (3.3%)	173 (4.2%)	147 (3.7%)	144 (3.9%)	137 (3.4%)	106 (2.3%)	64 (3.3%)	1,604 (3.5%)
Maternal treatment classification														
Adequate	68 (4.5%)	68 (3.5%)	111 (4.6%)	154 (5.2%)	138 (4.1%)	186 (4.9%)	209 (5%)	299 (7.3%)	268 (6.7%)	311 (8.3%)	409 (10.2%)	505 (11.1%)	183 (9.5%)	3,024 (6.6%)
Inadequate	779 (51.7%)	991 (51.5%)	1,224 (51.1%)	1,609 (54.1%)	1,790 (52.7%)	2,115 (55.6%)	2,204 (53.1%)	1,961 (47.9%)	1,859 (46.7%)	1,668 (44.7%)	1,683 (42%)	2,034 (44.8%)	909 (47.4%)	22,757 (49.5%)
Not performed	539 (35.8%)	740 (38.4%)	805 (33.6%)	960 (32.3%)	1,080 (31.8%)	1,106 (29.1%)	1,230 (29.6%)	1,343 (32.8%)	1,411 (35.5%)	1,291 (34.6%)	1,651 (41.2%)	1,637 (36.1%)	647 (33.8%)	15,683 (34.1%)
Unknown	120 (8%)	127 (6.6%)	257 (10.7%)	253 (8.5%)	386 (11.4%)	400 (10.5%)	508 (12.2%)	493 (12%)	442 (11.1%)	462 (12.4%)	266 (6.6%)	360 (7.9%)	178 (9.3%)	4,555 (9.9%)
Partner treatment status														
Yes	180 (12%)	210 (10.9%)	267 (11.1%)	364 (12.2%)	380 (11.2%)	491 (12.9%)	587 (14.1%)	946 (23.1%)	915 (23%)	962 (25.8%)	1,193 (29.8%)	1,511 (33.3%)	690 (36%)	9,053 (19.7%)
No	1,109 (73.6%)	1,498 (77.8%)	1,721 (71.8%)	2,178 (73.2%)	2,431 (71.6%)	2,718 (71.4%)	2,958 (71.3%)	2,290 (55.9%)	2,249 (56.5%)	1,958 (52.5%)	1,882 (46.9%)	2,009 (44.3%)	850 (44.3%)	28,522 (62%)
Unknown	217 (14.4%)	218 (11.3%)	409 (17.1%)	434 (14.6%)	583 (17.2%)	598 (15.7%)	606 (14.6%)	860 (21%)	816 (20.5%)	812 (21.8%)	934 (23.3%)	1,016 (22.4%)	377 (19.7%)	8,444 (18.3%)
Perinatal outcomes														
Live birth	1352 (89.8%)	1,698 (88.2%)	2,090 (87.2%)	2,565 (86.2%)	2,945 (86.8%)	3,323 (87.3%)	3,597 (86.7%)	3,488 (85.2%)	3,402 (85.5%)	3,201 (85.8%)	3,419 (85.3%)	3,901 (86.0%)	1,649 (86.0%)	39,786 (86.5%)
Deaths from CS	11 (0.7%)	19 (1.0%)	22 (0.9%)	32 (1.1%)	49 (1.4%)	42 (1.1%)	47 (1.1%)	41 (1.0%)	37 (0.9%)	43 (1.2%)	50 (1.2%)	36 (0.8%)	19 (1.0%)	485 (1.1%)
Deaths from other causes	8 (0.5%)	17 (0.9%)	30 (1.3%)	27 (0.9%)	21 (0.6%)	34 (0.9%)	37 (0.9%)	27 (0.7%)	28 (0.7%)	19 (0.5%)	33 (0.8%)	37 (0.8%)	21 (1.1%)	370 (0.8%)
Miscarriage	65 (4.3%)	90 (4.7%)	116 (4.8%)	143 (4.8%)	178 (5.2%)	189 (5.0%)	232 (5.6%)	310 (7.6%)	307 (7.7%)	288 (7.7%)	301 (7.5%)	350 (7.7%)	128 (6.7%)	2,861 (6.2%)
Stillbirth	45 (3.0%)	80 (4.2%)	91 (3.8%)	169 (5.7%)	150 (4.4%)	136 (3.6%)	157 (3.8%)	174 (4.2%)	142 (3.6%)	134 (3.6%)	156 (3.9%)	147 (3.2%)	73 (3.8%)	1,753 (3.8%)
Unknown	25 (1.7%)	22 (1.1%)	48 (2.0%)	40 (1.3%)	51 (1.5%)	83 (2.2%)	81 (2.0%)	56 (1.4%)	64 (1.6%)	47 (1.3%)	50 (1.2%)	65 (1.4%)	27 (1.4%)	764 (1.7%)

**Source: SINAN:** *Sistema de Informação de Agravos de Notificação*, DATASUS/MS.

Between 2011 and 2023, most maternal syphilis diagnoses were made during prenatal care, increasing from 51.9% in 2011 to 67.5% in 2023. Diagnoses at delivery or curettage decreased from 39.2% to 27.6%, whereas post-delivery diagnoses consistently remained below 6.5%, reaching 1.7% by 2023. The percentage of cases in which testing was not performed or the timing was unknown remained below 3.5%. Regarding prenatal care coverage, the proportion of women reporting prenatal care increased from 74% in 2011 to 83.6% in 2023. Conversely, those without prenatal care decreased from 23% to 13%. Cases with unknown prenatal care status fluctuated between 2.3% and 4.2% (Table 1).

The proportion of inadequate maternal treatment remained high, ranging from 51.1% to 55.6% between 2011 and 2017, before declining to 42.0%-47.4% in later years. The number of untreated cases remained substantial, varying from 29.1% to 41.2%. Despite some improvements, adequate treatment remained low, peaking at only 11.1% in 2022. Partner treatment increased from 12% in 2011 to 36% in 2023, whereas the proportion of untreated partners declined from 73.6% to 44.3%. However, cases with unknown partner treatment status remained consistently high (11.3%-23.3%) (Table 1).

In 2016, the World Health Organization launched a global initiative to eliminate congenital syphilis, with the goal of reducing incidence to ≤50 cases per 1,000 live births in 80% of countries by 2030^7^. In our study, the incidence of congenital syphilis continued to increase, peaking at 8.9 cases per 1,000 live births in 2022, while mortality also rose, particularly after 2012. In Rio Grande do Norte (Northeast Brazil), a study conducted between 2008 and 2018 reported an increased risk of congenital syphilis of up to 2.65 times, with an incidence rate of 7.91 cases per 1,000 live births^8^. Similarly, an epidemiological study in Tocantins (North Region) between 2007 and 2015 reported 1,029 cases, with mean incidence rising from 3.1 per 1,000 live births in 2007 to 9.8 per 1,000 in 2015, representing a 216.1% increase^9^. Together, these studies [8,9] confirm the increasing burden of congenital syphilis in different Brazilian regions, particularly after 2010.

In our study, most maternal syphilis diagnoses were made during the prenatal care period between 2011 and 2023. Similarly, in a study conducted in China involving 15,884 women who gave birth in 2013, 79.1% of infected women were diagnosed during prenatal care^10^. In the state of Santa Catarina (South Brazil), from 2007 to 2017, 65.4% of congenital syphilis diagnoses were also made during prenatal care^11^. 

The proportion of women who received inadequate maternal treatment was high in this study. In Ceará (Northeast Brazil), between 2000 and 2009, 2,930 cases were reported, with inadequate treatment predominating, particularly in 2005, when 56.8% of women received inadequate treatment^12^. Our study also found a significant increase in mortality rates after 2012. A study conducted in six Brazilian states and the Federal District (2012-2017) reported miscarriage outcomes ranging from 2.2% in Amazonas to 5.6% in Ceará, while stillbirths ranged from 3.3% to 10.9%^13^.

Regarding partner treatment, our findings are consistent with those in other regions. In Palmas (Tocantins), 204 cases were identified (5.6 per 1,000 live births), and among women who received prenatal care, 83.0% of partners remained untreated^14^. 

This study had some limitations worth noting. First, the use of secondary data from national surveillance systems is subject to underreporting, reporting delays, and potential inconsistencies in data quality across municipalities over time. The presence of missing or unknown information, particularly regarding key variables such as maternal treatment adequacy and partner treatment status, may compromise the accuracy of proportions and limit the interpretation of temporal trends. In addition, because ecological studies are based on aggregated data, individual-level associations or causal inferences cannot be established. Finally, the 2023 dataset was partial and included only descriptive percentages, which may not reflect the complete annual profile for that year.

In conclusion, this historical analysis revealed persistent deficiencies in congenital syphilis prevention, despite increased access to prenatal care. The rising incidence and mortality, coupled with persistently inadequate maternal and partner treatment, underscore the urgent need for improved screening, timely antibiotic administration, and partner engagement strategies.

## Data Availability

Research data is available in the repository https://portalsinan.saude.gov.br
